# Nomogram Based on Cytokines for Cardiovascular Diseases in Xinjiang Kazakhs

**DOI:** 10.1155/2019/4756295

**Published:** 2019-05-05

**Authors:** Lei Mao, Xianghui Zhang, Yunhua Hu, Xinping Wang, Yanpeng Song, Jia He, Wenwen Yang, Jiaolong Ma, Yizhong Yan, Lati Mu, Jingyu Zhang, Kui Wang, Heng Guo, Rulin Ma, Shuxia Guo

**Affiliations:** ^1^Department of Public Health, Shihezi University School of Medicine, Shihezi, Xinjiang 832000, China; ^2^The First Affiliated Hospital of Shihezi University Medical College, Shihezi, Xinjiang 832000, China; ^3^Department of Pathology and Key Laboratory of Xinjiang Endemic and Ethnic Diseases (Ministry of Education), Shihezi University School of Medicine, Shihezi, Xinjiang 832000, China

## Abstract

**Background:**

This study involved the development of a predictive 5-year morbidity nomogram for cardiovascular diseases (CVD) in Xinjiang Kazakhs based on cytokine levels.

**Methods:**

The nomogram was based on a baseline survey of the town of Nalati in the Kazakh Autonomous Prefecture of Xinjiang from 2009 to 2013. By 2016, we had monitored 1508 people for a median time of 5.17 years and identified CVD events in the study population by collecting case information from local hospitals. The study population was divided into the training (*n* = 1005) and validation cohorts (*n* = 503) in a 2 : 1 ratio. The area under the receiver operating characteristic curve (AUC) was used to verify the predictive accuracy of the nomogram. The result was assessed in a validation cohort.

**Results:**

At the end of the study, the incidence of CVD in Xinjiang Kazakhs was found to be 11.28%. We developed a new nomogram to predict the 5-year incidence of CVD based on age, interleukin-6 (IL-6), and adiponectin (APN) levels, diastolic blood pressure, and dyslipidemia. The AUC for the predictive accuracy of the nomogram was 0.836 (95% confidence interval: 0.802–0.869), which was higher than that for IL-6 and APN. These results were supported by validation studies.

**Conclusions:**

The nomogram model can more directly assess the risk of CVD in Kazakhs and can be used for CVD risk assessment.

## 1. Introduction

Cardiovascular diseases (CVD) are among the most common causes of death worldwide, and over three-quarters of CVD deaths occur in low- and middle-income countries [1]. CVD has become the leading cause of death in 27 provinces and cities in China, which is one of the most populous developing countries. The economic burden caused by CVD should not be underestimated [2]. The development of atherosclerosis is the pathophysiological basis of CVD. Free fatty acids (FFAs) [3], high-sensitivity C-reactive protein (hs-CRP) [4], adiponectin (APN) [5], insulin (INS) [6], and interleukin-6 (IL-6) [7] can induce atherosclerosis through arterial inflammation. Smoking, drinking, and other adverse lifestyle-related risk factors eventually lead to the development of CVD. Therefore, a complete understanding of the risk factors of CVD and the identification and control of risk factors in high-risk groups can help prevent the development of CVD and reduce the burden of disease.

The use of risk factors to construct a CVD risk assessment model for the estimation of the probability of CVD is effective. The classic Framingham study has predicted the risk of CVD in native white Americans over a decade; the risk prediction scale developed from this study is used to evaluate common clinical risk factors [8]. Chinese researchers have used this study as a reference for a prospective cohort study in 11 provinces and cities to investigate hypertension, hyperlipidemia, diabetes, and obesity, which are independent risk factors for CVD in the Han Chinese population [9]. The intermediate inflammatory and lipid factors in the CVD cascade can reflect premorbid status. The proximal or intermediate risk factors can be used to improve the predictive ability of a CVD model. In a multiethnic country, the prevalence of CVD varies among different ethnic groups and an established CVD risk prediction model cannot be uniformly applied to all groups. Therefore, based on the characteristics of different nationalities and population groups, a CVD model can be applied to a study population.

The population in our study included Kazakhs living in the remote rural areas of Western China. Kazakhs gather in mountainous pastures in Northern Xinjiang and have a mainly nomadic existence. Xinjiang has the second largest population of this local minority. Previous studies found that the CVD-related risk factors in the Kazakh population are hypertension, obesity, and metabolic syndrome (MetS), which have high prevalence rates [10–12]. Their levels of FFAs, APN, INS, IL-6, and hs-CRP differed from those of other ethnic groups, and their risk of CVD is higher [13–16]. In our study, cytokine levels combined with common risk factors for CVD were used to construct a nomogram for use in the Xinjiang Kazakh rural population and to evaluate the incidence and risk of CVD in these individuals. The data can be used for early screening of CVD in rural areas.

## 2. Materials and Methods

### 2.1. Study Population

The survey was conducted from 2009 to 2013 in Nalati town, Xinjiang Kazakh Autonomous Region, which is 4407 km (2739 miles) from Beijing. We successfully interviewed 1771 local Kazakh individuals aged 18 years or older who had resided in the village for at least 6 months. By the end of 2016, a total of 1508 Kazakh inhabitants with complete data were enrolled in the study and were followed up for a median of 5.17 years. We identified the CVD cases in this population using medical records from the People's Hospital of Xinjiang Xinyuan County and Traditional Chinese Medicine Hospital of Xinjiang Xinyuan County. Each participant provided written informed consent and completed questionnaires. Evaluation included disease history, height, weight, waist circumference, hip circumference, and blood pressure. Blood pressure was measured three times in each subject using a mercury sphygmomanometer, and the average value was calculated. The study was approved by the Institutional Ethics Review Board of the First Affiliated Hospital of Shihezi University (IERB no. SHZ2010LL01).

Subjects with complete information were randomly assigned in a 2 : 1 ratio into the training cohort (*n* = 1005) to develop a nomogram and the internal validation cohort (*n* = 503) to evaluate the predictive models.

### 2.2. Definitions

CVD was identified as follows: (1) hospitalizations for CVD at follow-up, coronary intervention (cardiac catheterization or coronary bypass), angina (or nitroglycerin after cohort study), and CVD death (ICD9: codes 390-495); (2) according to the hospital medical records, hospitalization was due to the following reasons: coronary artery atherosclerosis, coronary heart disease, unstable angina, myocardial infarction, heart failure, stroke, transient cerebral ischemia, and peripheral vascular disease (abdominal aortic aneurysm, peripheral vascular surgery, or carotid endarterectomy); and (3) the CVD events were recorded according to the hospitalization records and questionnaires. If two or more events of the same class of subjects occur, then the first occurrence was the outcome event.

Based on the diagnostic criteria recommended by the IDF, participants were considered to have MetS if they had abdominal obesity (WC ≥ 80 cm in women and ≥90 cm in men) and showed two or more of the following: (1) hypertriglyceridemia (≥150 g/dL); (2) low HDL-C (<50 mg/dL in women and <40 mg/dL in men); (3) high blood pressure (systolic blood pressure ≥ 130 mmHg or diastolic blood pressure ≥ 85 mmHg); and (4) high fasting glucose (≥100 mg/dL).

Based on the China Adult Dyslipidemia Prevention Guide (2007) [15], dyslipidemia was defined as the presentation of any one or more of the following four items: (1) TG ≥ 2.26 mmol/L (200 mg/dL); (2) HDL − C < 1.04 mmol/L (40 mg/dL); (3) LDL − C ≥ 4.14 mmol/L (160 mg/dL); and (4) TC ≥ 6.22 mmol/L (240 mg/dL).

### 2.3. Methods

Total cholesterol (TC), triglycerides (TG), high-density lipoprotein cholesterol (HDL-C), low-density lipoprotein cholesterol (LDL-C), and fasting blood glucose were analyzed using an automatic biochemical analyzer (Olympus AU2700, Olympus Diagnostics, Hamburg, Germany) in the clinical laboratory.

FFA levels were determined by a colorimetric assay using kits purchased from Randox Laboratories Ltd. (Shanghai, China). ELISA kits for detecting the levels of INS, hs-CRP, IL-6, and APN were purchased from Elabscience Biotechnology (Wuhan, China).

### 2.4. Statistical Analysis

All statistical analyses were preformed using SPSS version 19.0, R (http://www.r-project.org/, The R Foundation), and EmpowerStats (http://www.empowerstats.com, X&Y Solutions Inc., Boston, MA). Baseline comparisons between the two cohorts were performed using Student's *t*-test or the Mann-Whitney test for continuous variables where appropriate and chi-square tests for categorical variables. The hs-CRP, IL-6, and APN levels displayed a positively skewed distribution. After logarithmic (Lg_10_) transformation, the data were approximately normally distributed with a geometric mean (*G*) and corresponding 95% confidence intervals (95% CI) for hs-CRP, IL-6, and APN. Multivariable Cox proportional hazards regression analysis was used to identify independent risk factors through stepwise selection. A nomogram was established based on the results of multivariable analysis [17]. Discriminative ability was measured using an area under the receiver operating characteristic (ROC) curve (AUC) and a calibration curve by comparing the probability using the predictive nomogram to actually observe CVD probability. An ROC curve was drawn to determine the optimal threshold of the nomogram. A 2-sided *P* value < 0.05 was considered statistically significant.

## 3. Results

### 3.1. The Baseline Characteristics of the Kazakhs

At the end of the study, 203 CVD events (CHD, myocardial infarction, stroke, etc.) were diagnosed, with a prevalence of 13.46%. In the training cohort, the average median age was 46.02 years old and 448 (44.6%) were male, of which 13.5% of the Kazakhs (*n* = 136) were diagnosed CVD at the end of the follow-up. In the validation cohort, the average median age was 44.99 years old and 214 (42.5%) were male, of which 13.3% of the Kazakhs (*n* = 67) were diagnosed CVD at the end of the follow-up. The baseline characteristics between the training cohort and the internal validation were not significantly different (Table 1).

### 3.2. Hazard Analysis in the Training Cohort

Univariate analysis showed that age, MetS, INS, Lg(hs-CRP), Lg(IL-6), FFAs, Lg(APN), SBP, DBP, body mass index (BMI), smoking, diabetes, family history of hypertension, and dyslipidemia were differed significantly between CVD and non-CVD cases. On Cox regression analysis, Lg(IL-6) (relative risk (HR) 1.252; 95% confidence interval (CI) 1.059–1.481), Lg(APN) (HR 0.075; 95% CI 0.588–0.845), DBP (HR 1.011; 95% CI 1.002–1.019), and dyslipidemia (HR 1.485; 95% CI 1.090–2.024) were independent risk factors for CVD patients in the Kazakh population. (Table 2).

### 3.3. Usage of Nomogram

**Figure 1:**
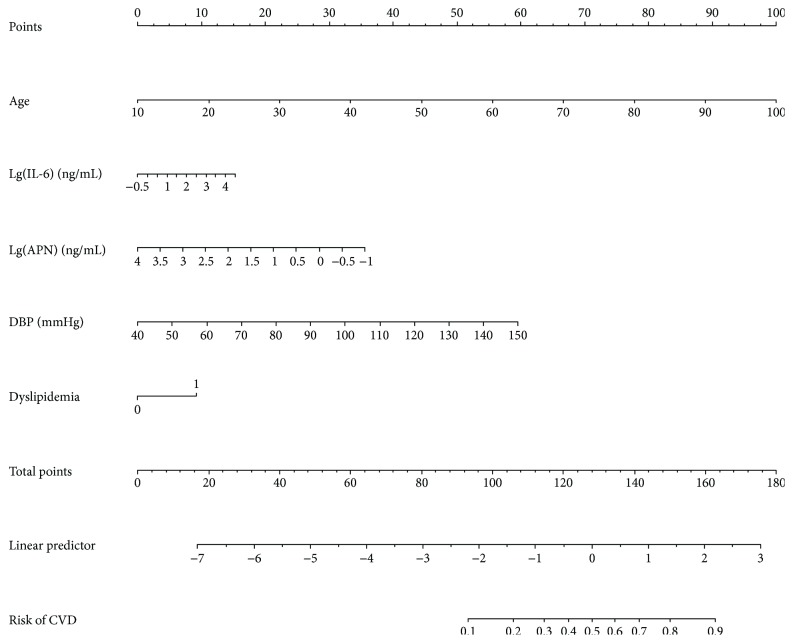
Nomogram to predict the risk of CVD in the Kazakhs. Draw an upward vertical line from each variable axis to the Points bar to get the points of each variable. Based on the sum of each variable points, draw a downward vertical line from Total points axis to calculate Risk of CVD bar.

By taking a Kazakhs who is 50 years old as an example, drawing an upward vertical line from the age variable axis to the Point bar gets a point of 45. If the IL-6 of this Kazakh is 100 ng/mL, the point corresponding to the IL-6 variable is 7.5. If the APN of this Kazakh is 3.16 ng/mL, the point corresponding to the APN variable is 25. If the DBP of a Kazakh is 90 mmHg, the corresponding point is 27.5. And if he/she has dyslipidemia, the corresponding point is 10. The total points equal to 115 (45 + 7.5 + 25 + 27.5 + 10). So, the risk of CVD is 30% when we draw a straight line from the total point axis to the risk of CVD axis (Figure 1).

### 3.4. Nomogram for CVD Prediction in Training Cohort

**Figure 2:**
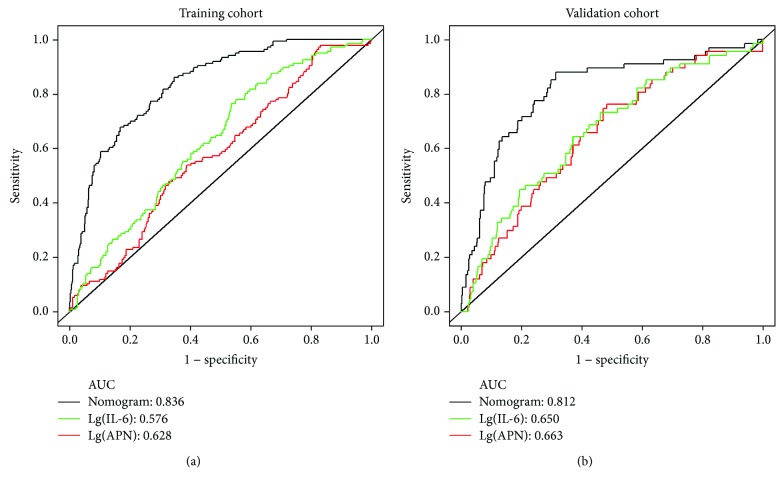
ROC curve of the nomogram and other models to predict the risk of CVD in Kazakhs. (a) ROC curve in training cohort. (b) ROC curve in validation cohort.

The prognosis for the independent risk factors of age, IL-6, ANP, DBP and dyslipidemia were incorporated into the nomogram. The AUC of the nomogram for CVD prediction was 0.836 (0.802-0.869), while the AUC was 0.576 (0.527-0.625) for Lg(IL-6) and 0.628 (0.582-0.675) for Lg(APN), respectively (Figure 2(a)). The difference was statistically significant between the nomogram and Lg(IL-6) (*P* < 0.001) and between the nomogram and Lg(APN) (*P* < 0.001). In the training cohort, the Youden's index of nomogram was 0.514. (Sen = 86.03% and Spe = 65.36%) and an optimal cutoff of -2.293 (Table 3).

### 3.5. Nomogram for CVD Prediction in Validation Cohort

Using CVD as the end point, the AUC was 0.812 (0.753-0.871) for the nomogram, 0.650 (0.582-0.719) for the Lg(IL-6), and 0.663 (0.593-0.733) for Lg(APN), respectively (Figure 2(b)). The difference was statistically significant between the nomogram and Lg(IL-6) (*P* < 0.001) and between the nomogram and Lg(APN) (*P* < 0.001). In validation cohort, the nomogram had a Youden index value of 0.567 (Sen = 88.06% and Spe = 68.58%) and an optimal cutoff of -2.307 (Table 3).

## 4. Discussion

CVD is a life-threatening noncommunicable disease with high morbidity. In our study, the incidence of CVD was 13.46%; this was higher than in other reports [18, 19]. The prevalence of hypertension in the Xinjiang Kazakh population is high, and the potential population with CVD is large. Therefore, strengthening the primary prevention of CVD in this ethnic groups and reducing the risk and disease burden are necessary.

The prevalence of risk factors, including age, MetS, hypertension, obesity, smoking, diabetes, family history of hypertension, and dyslipidemia, was greater in Kazakh patients with CVD than in those without CVD. Many studies have reported that the prevalence of CVD is associated with age, hypertension, diabetes, smoking, and obesity [20–24]. Insulin resistance, lipokine levels, and inflammation can also lead to adverse cardiovascular events [25]. Abnormal secretion of INS can lead to resistance, which plays a key role in atherosclerotic plaque formation [26]. Eddy et al. [27] suggested that the prevention of INS resistance in nondiabetic adults could prevent approximately 42% of myocardial infarctions and reduce the risk of CVD. Our study found that the levels of INS in the CVD group were significantly higher than those in the non-CVD group; moreover, INS resistance in the CVD group was more severe and the risk of CVD in Kazakhs was high. The decrease in INS sensitivity was related to the increase in serum FFA levels and the decrease in APN levels. This study also found that the FFA levels in the CVD group were higher than those in the non-CVD group, whereas the APN levels were lower than those in the non-CVD group. Elevated levels of FFAs can inhibit endogenous NO activity, affect vascular endothelial function, and aggravate the degree of atherosclerosis. However, APN is a hormone secreted by adipocytes, which can increase fatty acid oxidation, reduce FFA content, and improve lipid metabolism, thereby playing an antiatherosclerotic role. Therefore, the treatment of Kazakhs with elevated plasma FFA and decreased APN levels may be helpful for the prevention and treatment of CVD. hs-CRP and IL-6, as mediators and markers of atherosclerotic thrombosis, play an important role in the development of CVD [28]. The levels of hs-CRP and IL-6 in the CVD group were higher than those in the non-CVD group, consistent with the findings in previous studies [29, 30]. This suggested that the levels of hs-CRP and IL-6 were elevated, the population may be present in the pre-onset phase of CVD. Our study also found that the prevalence of MetS, diabetes, and dyslipidemia was greater in the population with CVD than in those without CVD. Multiple chronic diseases were clustered in some individuals, possibly because Kazakhs live in remote rural areas in Western China, lack health knowledge, have a unique high-fat and high-carbohydrate diet, and have chronic exposure to CVD risk factors. Thus, health education is needed for Kazakhs, in addition to the construction of a CVD predictive model, identification of high-risk groups, control of risk factors, and reduction in the incidence of chronic diseases, such as CVD.

Our CVD nomogram has 5 components: age, IL-6 and APN levels, DBP, and dyslipidemia, with a predictive ability higher than that of the Framingham study (AUC approximately 0.80) [31] and the simple model for a 10-year risk assessment program for ischemic cardiovascular disease in Chinese (AUC = 0.796) [32]. All previous models included common CVD risk factors but lacked information on cytokines, such as IL-6 and APN. Changes in IL-6 and APN levels can reflect both the severity of atherosclerosis and thrombosis and the dynamic process of CVD. This study found that increased plasma IL-6 levels and decreased APN levels were risk factors for CVD in Kazakhs. The respective AUC values predictive of CVD were 0.576 and 0.628 and were significantly lower than those in the CVD nomogram. The plasma levels of IL-6 and APN can be used to predict CVD. The plasma levels of APN as a protective factor against atherosclerosis can also be used to predict the development of CVD [33]. However, IL-6 affected atherosclerosis through synergistic action with other inflammatory factors, subsequently leading to CVD [34]. Dyslipidemia is an independent risk factor for CVD, and its prevalence in Kazakhs is significantly higher than in the rest of China [35]. This study also found that the risk of CVD in Kazakh patients with dyslipidemia was 1.485 times higher than those without dyslipidemia, suggesting that the risk of CVD was increased by the presence of dyslipidemia. Although the indicators of dyslipidemia were included in most CVD predictive models, the weighting was different among models, suggesting the need to construct a targeted CVD risk model to maximize the usefulness of CVD risk factors.

Validation sampling is important for judging the applicability of a CVD model. By using a validation sample to evaluate predictive ability, we can accurately assess predictive efficacy and optimize predictive ability. Through the use of a validation sample for the CVD nomogram, the AUC was found to be 0.812 (95% CI: 0.753–0.871). This shows that the CVD nomogram can be applied locally to Kazakhs. Many studies have used models constructed by others to predict disease prevalence; these have “local advantages” and poor extrapolation ability [36]. This study constructed a CVD nomogram according to the characteristics of the Kazakh population. The model predicted the risk of CVD according to common and intermediate risk factors, including cytokine levels. This is a convenient and direct method for the determination of the risk of CVD.

This study has three advantages. First, based on the characteristics of local Kazakh disease prevalence, cytokines and common risk factors were used as predictors to construct a risk model for CVD in the Kazakh population. Second, we compared the aggregation characteristics of glucose and lipid (FFAs, INS, and APN) metabolism and inflammatory markers (IL-6 and hs-CRP) in patients with and without CVD in the Kazakh population. We discussed the risk factors for CVD and used these to construct a targeted CVD risk model. Finally, this study also verified the applicability of the model to a population group. Therefore, the CVD risk nomogram constructed in this study can be used for early screening in primary medical institutions to estimate the risk of CVD in local Kazakh populations and reduce its prevalence in rural areas of Xinjiang by controlling risk factors.

This study has some limitations. First, the included indicators do not fully cover all risk factors for CVD, thereby resulting in a limited predictive power of the model. Second, this study and CVD model were based on the characteristics of the Kazakh population. Whether the model can be applied to other ethnic groups requires further investigation. The small sample size of this study is mainly due to the nomadic lifestyle of Kazakhs, which prevented the follow-up of all subjects. Therefore, further research can supplement follow-up information, increase the sample size, and select more accurate indicators to reflect the risk of CVD, thereby improving the predictive ability of the model.

## Figures and Tables

**Table 1:** Baseline characteristics of the training and validation cohorts.

Variables	Training cohort (791)	Validation cohort (264)	*P* value
Sex, male *n* (%)	338 (42.7)	109 (41.3)	0.681
Age (years)	47.37 ± 13.48	46.64 ± 12.66	0.647
MetS (%)	149 (18.8)	56 (21.2)	0.398
FFAs (mmol/L)	0.69 ± 0.56	0.71 ± 0.66	0.483
INS (ng/mL)	2.39 ± 1.26	2.40 ± 1.27	0.932
hs-CRP (pg/mL)	2.72 ± 0.60	2.73 ± 0.61	0.747
IL-6 (ng/mL)	34.52 (17.96-70.30)	33.53 (18.69-76.01)	0.655^#^
APN (ng/mL)	22.82 ± 17.59	23.87 ± 17.57	0.400
CVD (%)	94 (11.9)	25 (9.5)	0.283
Smoking (%)	274 (34.6)	86 (32.6)	0.540
Alcohol drinking (%)	96 (12.1)	24 (9.1)	0.177
Hypertension (%)	275 (34.8)	97 (36.7)	0.561
Family history of hypertension (%)	265 (33.5)	103 (39.02)	0.104
Diabetes (%)	29 (3.7)	16 (6.1)	0.096
Dyslipidemia (%)	309 (39.1)	101 (38.3)	0.816

^#^Mann-Whitney test.

**Table 2:** Baseline characteristics of CVD and non-CVD patients in the training group.

Variables	CVD (*n* = 136)	Non-CVD (*n* = 869)	Univariate analysis	Multivariate analysis			
			*P* value	*β*	*P* value	HR	95% CI
Sex, male *n* (%)	53 (39.0)	395 (45.5)	0.157				
Age (years)	58.69 ± 11.14	44.13 ± 12.49	<0.001				
MetS (%)	46 (33.8)	208 (23.9)	0.014				
INS (ng/mL)^#^	14.22 (7.66-25.38)	9.58 (5.42-20.67)	0.001				
Lg(hs-CRP) (pg/mL)	2.73 ± 0.86	2.26 ± 1.01	<0.001				
Lg(IL-6) (pg/mL)	1.81 ± 0.77	1.65 ± 0.78	0.021	0.225	0.009	1.252	1.059-1.481
NEFA (mmol/L)^#^	0.59 (0.40-1.10)	0.48 (0.33-0.76)	<0.001				
Lg(ADP) (ng/mL)	1.32 ± 0.69	1.71 ± 0.92	<0.001	-0.350	<0.001	0.075	0.588-0.845
SBP (mmHg)	149.54 ± 31.28	126.55 ± 20.32	<0.001				
DBP (mmHg)	93.74 ± 17.39	81.01 ± 12.63	<0.001	0.011	0.011	1.011	1.002-1.019
BMI	25.42 ± 4.83	23.58 ± 3.69	<0.001				
Current smoker (%)	61 (44.9)	224 (28.1)	<0.001				
Alcohol drinking (%)	20 (14.7)	88 (10.1)	0.109				
Diabetes (%)	8 (5.9)	22 (2.5)	0.033				
Family history of hypertension (%)	54 (39.7)	237 (27.3)	0.003				
Dyslipidemia (%)	46 (33.8)	211 (24.3)	0.018	0.359	0.012	1.485	1.090-2.024

Note: ^#^Mann-Whitney test. MetS: metabolic syndrome; INS: insulin; hs-CRP: high-sensitivity C-reactive protein; IL-6: interleukin 6; NEFA: nonesterified fatty acids; ADP: adiponectin; SBP: systolic blood pressure; DBP: diastolic blood pressure; BMI: body mass index.

**Table 3:** The results of ROC curves of training cohort and validation cohort in the Kazakhs.

	Variables	Cutoff	Sen (%)	Spe (%)	Youden's index	AUC (95% CI)	*P* value
Model 1	Lg(IL-6)	1.703	47.79	67.09	0.149	0.576 (0.527-0.625)	*P* < 0.001
	Lg(APN)	1.561	76.47	46.49	0.230	0.628 (0.582-0.675)	*P* < 0.001
	Nomogram	-2.293	86.03	65.36	0.514	0.836 (0.802-0.869)	*P* < 0.001

Model 2	Lg(IL-6)	1.495	76.12	51.83	0.280	0.650 (0.582-0.719)	*P* < 0.001
	Lg(APN)	1.470	73.13	53.90	0.270	0.663 (0.593-0.733)	*P* < 0.001
	Nomogram	-2.307	88.06	68.58	0.567	0.812 (0.753-0.871)	*P* < 0.001

Model 1, training cohort. Model 2, validation cohort. Sen: sensitivity; Spe: specificity.

## Data Availability

The data used to support the findings of this study are available from the corresponding author upon request.

## References

[1] Chen W. W., Sui H., Ma L. Y. (2016). Current situation and progress of prevention and treatment of cardiovascular and cerebrovascular diseases in China. *Prevention and Treatment of Cardio-Cerebral-Vascular Disease*.

[2] Zhang X. F., Hu D. Y., Ding R. J., Wang H. C., Yan L. X. (2012). Status and trend of cardio-cerebral-vascular diseases mortality in China:data from national disease surveillance system between 2004 and 2008. *Chinese Journal of Cardiology*.

[3] Chen F., Gao C. Z. (2010). Relationship between intima-media thickness and free fatty acids in hypertensive patients with metabolic syndrome. *Practical Journal Of Cardiac Cerebral Pneumal And Vascular Disease*.

[4] Zhou X., Li S. X. (2016). Changes and clinical significance of serum high sensitive C reactive protein and fibrinogen in patients with carotid atherosclerosis. *Chronic Pathematology Journal*.

[5] Gao J., Qu X. B. (2008). Progress of research on association between adiponectin and atherosclerosis. *Progress in Modern Biomedicine*.

[6] Yang B., Wang G. Y., Chen B., Chen L. (2007). Relationship between hyperglycemia and carotid atherosclerosis in Tibetan population. *Chinese Journal of Cardiovascular Rehabilitation Medicine*.

[7] Chen C. X., Lu Y. K., Bao Y. Z. (2011). Relationship between severity of carotid atherosclerotic plaque and serum levels of CRP, IL-6 and TNF-*α*. *Shandong Medical Journal*.

[8] Fox C. S., Evans J. C., Larson M. G., Kannel W. B., Levy D. (2004). Temporal trends in coronary heart disease mortality and sudden cardiac death from 1950 to 1999: the Framingham Heart Study. *Circulation*.

[9] Wu Z. S., Yao C. H., Zhao D. (1999). Prospective study of cardiovascular disease in 11 provinces and cities. I. Relationship between risk factors and cardiovascular disease. *Journal of Clinical Radiology*.

[10] Zhang X. H., Yan Y. Z., Jia H. E. (2017). Evaluation of screening indices for metabolic syndrome in adult Kazakh in Xinjiang. *Chinese Journal of Hypertension*.

[11] Ma X. J., Zhang M., Guo S. X. (2013). Prevalence of hypertension in Uygur, Kazakh and Han populations in rural areas of Xinjiang. *Chinese Journal of Hypertension*.

[12] He J., Guo H., Ding Y. S. (2013). Epidemiological study on overweight and obesity among rural adult residents in Hazakh, Uygur and Han populations in Xinjiang. *Chinese Journal of Epidemiology*.

[13] Guo S. X., Yang Z. M., Zhang J. Y. (2010). Relationship between hypertension and free fatty acids and insulin resistance in Kazakh and Han nationality in Xinjiang. *Chinese Journal of Hypertension*.

[14] Wang Z., Tian Y. J., Guo S. X. (2007). Comparison of plasma adiponectin levels in essential hypertension patients between Kazakan and Han nationalities. *Journal of Clinical Rehabilitative Tissue Engineering Research*.

[15] Sun K., Chang X. Y., Wang X. L., Yin L., Zhang C., Chen X. (2013). Comparison research of lipid metabolism and insulin resistance in type 2 diabetes mellitus of Han people and Kazakh lived in Xinjiang. *China Journal of Modern Medicine*.

[16] Zhang Q. (2010). *Correlation between Serum Levels of Interleukin-6 and High-Sensitivity C-Reactive Protein and Hypertension in Xinjiang Kazakh and Han Nationality*.

[17] Zhang Z., Kattan M. W. (2017). Drawing nomograms with R: applications to categorical outcome and survival data. *Annals of Translational Medicine*.

[18] Hoevenaar-Blom M. P., Nooyens A. C. J., Kromhout D. (2012). Mediterranean style diet and 12-year incidence of cardiovascular diseases: the EPIC-NL cohort study. *PLoS One*.

[19] Panagiotakos D. B., Pitsavos C., Chrysohoou C., Skoumas I., Stefanadis C., ATTICA Study (2008). Five-year incidence of cardiovascular disease and its predictors in Greece: the Attica study. *Vascular Medicine*.

[20] Li H. Y., Chen Y., Wang Q. H., Zhong Y. M., Zheng M., Xun C. Z. (2017). Epidemiological characteristics of cardiovascular and cerebrovascular diseases in Luquan County of Yunnan Province in 2013. *Chinese Journal of Prevention and Control of Chronic Diseases*.

[21] Cai Y., Cui H., Fan L. (2015). Prevalence of cardiovascular and cerebrovascular diseases in army old male hypertensive patients in Beijing. *Chinese Journal of Rehabilitation Theory and Practice*.

[22] Wang X. X., Xian T. Z., Jia X. F., Zhang L. N., Pan Q., Guo L. (2017). Nomogram analysis of the influencing factors of cardiovascular and cerebrovascular diseases in patients with type 2 diabetes mellitus. *Chinese Journal of Cardiovascular Medicine*.

[23] Keto J., Ventola H., Jokelainen J. (2016). Cardiovascular disease risk factors in relation to smoking behaviour and history: a population-based cohort study. *Open Heart*.

[24] Zhou B. F. (2002). Effect of body mass index on all-cause mortality and incidence of cardiovascular diseases—report for meta-analysis of prospective studies open optimal cut-off points of body mass index in Chinese adults. *Biomedical & Environmental Sciences*.

[25] Ormazabal V., Nair S., Elfeky O., Aguayo C., Salomon C., Zuñiga F. A. (2018). Association between insulin resistance and the development of cardiovascular disease. *Cardiovascular Diabetology*.

[26] Min J., Weitian Z., Peng C. (2016). Correlation between insulin-induced estrogen receptor methylation and atherosclerosis. *Cardiovascular Diabetology*.

[27] Eddy D., Schlessinger L., Kahn R., Peskin B., Schiebinger R. (2009). Relationship of insulin resistance and related metabolic variables to coronary artery disease: a mathematical analysis. *Diabetes Care*.

[28] Dong X. L., Li L., Liu G. Z. (2000). Application of d-two mer and its antibodies in cardiovascular diseases. *Journal of Capital University of Medical Sciences*.

[29] Sentürk T., Cordan J., Baran I. (2007). Procalcitonin in patients with acute coronary syndrome: correlation with high-sensitive C-reactive protein, prognosis and severity of coronary artery disease. *Acta Cardiologica*.

[30] Hashmi S., Zeng Q. T. (2006). Role of interleukin-17 and interleukin-17-induced cytokines interleukin-6 and interleukin-8 in unstable coronary artery disease. *Coronary Artery Disease*.

[31] de la Iglesia B., Potter J. F., Poulter N. R., Robins M. M., Skinner J. (2011). Performance of the assign cardiovascular disease risk score on a UK cohort of patients from general practice. *Heart*.

[32] Wu Y., Liu X., Li X. (2006). Estimation of 10-year risk of fatal and nonfatal ischemic cardiovascular diseases in Chinese adults. *Circulation*.

[33] Iwashima Y., Horio T., Kumada M. (2006). Adiponectin and renal function, and implication as a risk of cardiovascular disease. *The American Journal of Cardiology*.

[34] Wang G. Y., Kong F. L., Xu J., Pu X. Q., Wang K., Zhang Y. H. (2015). A cohort study of the association between IL-6, No and the incidence of stroke among Mongolian people. *Modern Preventive Medicine*.

[35] Ma R. L., Guo S. X., Li Y. (2012). Prevalence of dyslipidemia and its influencing factors in Kazakh adults. *Chinese Journal of Public Health*.

[36] Chen J. H., Wu H. Y., He K. L., He Y., Qin Y. H. (2010). Establishment of the prediction model for ischemic cardiovascular disease of elderly male population under current health care program. *Chinese Journal of Epidemiology*.

